# Pluronic F127 self-assembled MoS_2_ nanocomposites as an effective glutathione responsive anticancer drug delivery system

**DOI:** 10.1039/c9ra04249k

**Published:** 2019-08-15

**Authors:** Adhisankar Vadivelmurugan, Rajeshkumar Anbazhagan, Vinothini Arunagiri, Juin-Yih Lai, Hsieh-Chih Tsai

**Affiliations:** Graduate Institute of Applied Science and Technology, National Taiwan University of Science and Technology Taipei 106 Taiwan h.c.tsai@mail.ntust.edu.tw +886-2-27303625; Advanced Membrane Materials Center, National Taiwan University of Science and Technology Taipei 106 Taiwan; R&D Center for Membrane Technology, Chung Yuan Christian University Chungli Taoyuan 320 Taiwan

## Abstract

In this study, bio-responsive polymeric MoS_2_ nanocomposites were prepared for use as a drug carrier for cancer therapy. Herein, we report the synthesis and demonstrate the self-assembly of pluronic F127 (PF127) on a cystamine–glutathione–MoS_2_ (CYS–GSH–MoS_2_) system, which can be used for GSH-triggered drug release under biological reducing conditions. The reduction-sensitive disulfide bond containing CYS was incorporated between the amphiphilic copolymer PF127 and GSH–MoS_2_ to achieve feasible drug release. Percent drug loading capacity and encapsulation efficiency were 51.3% and 56%, respectively. In addition, when the MoS_2_–GSH–CYS–PF127 nanocomposite was incubated in a GSH environment, the morphology of the nanocomposite tended to change, ultimately leading to drug release. The drug-loaded PF127–CYS–GSH–MoS_2_ polymeric nanocomposites efficiently released 52% of their drug content after 72 h of incubation in a GSH reduction environment. The HeLa cells treated with DOX loaded MoS_2_–GSH–CYS–PF127 showed 38% toxicity at drug concentration of 40 μg, which indicated that the successfully released of drug from carrier and caused the cell death. Further, fluorescence microscopy images of HeLa cells revealed the potential behavior of the MoS_2_–GSH–CYS–PF12 nanocomposite during the 2- and 4 h incubation periods; the nanocomposite was only found in the cytoplasm of HeLa cells. Interestingly, after 6 h of incubation, the drug was slowly released from the nanocomposite and could enter the nucleus as confirmed by fluorescence imaging of HeLa cells. Altogether, our synthesized PF127-coated MoS_2_ nanocomposite could be effectively adopted in the near future as a GSH-sensitive drug carrier.

## Abstract 2

In this study, bio-responsive polymeric MoS_2_ nanocomposites were prepared for use as a drug carrier for cancer therapy.

## Graphical abstract

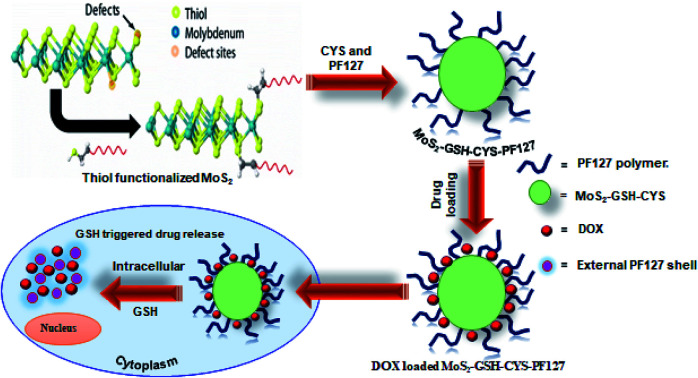

## 1. Introduction

In the past decade, there has been a growing interest for the construction of a remarkable reduction-responsive drug delivery system (DDS).^1^ Recently, various biomolecules and DDSs have been synthesized as stimuli-responsive nanocarriers toward light, pH, magnetic field, ultrasound, and redox potential by changing their surrounding environment. For example, Kim *et al.* reported that by changing the physicochemical properties of their delivery systems to cleave the disulphide linkage in the carrier to deliver the gene, the systems could be utilized to perform effective delivery and control cargo release at the target site.^2^ In gene delivery systems, stimuli-responsive carriers have shown great potential by overcoming many obstacles in cellular gene delivery such as cellular uptake, escape from endosomes, and cargo release of biomolecules at the targeted intracellular location.^3,4^ Generally, researchers have focused on multifunctional stimuli-responsive nanocarriers that can release drugs in response to internal or external stimuli such as pH,^5^ redox,^6^ temperature,^7^ enzyme,^8–10^ magnetic, and light^11–16^ in the environment. Such nanocarriers have recently been engineered as smart DDS.^17^ Simultaneous exposure to inherent or external stimuli-sensitive DDS activates activities that regulate drug release or adequately facilitate intracellular uptake or diffusion.^18^ Moreover, drug release following administration may be achieved *via* structurally modifying the microcontroller class of DDS transporting the drug or breaking down the chemical components of the nanocarrier. The unique biochemistry of various engineered stimuli-sensitive DDS under specific conditions can result in specific temporal and spatial DDSs.^19–21^ Nanocarriers are commonly designed to respond to a single external stimuli that activates drug release.^22,23^ Usually, these include intracellular GSH,^24–27^ ROS,^28,29^ and lysosomal activity.^30^ For GSH-triggered drug release, GSH acts as a reducing agent in the intracellular region by cleaving the sensitive disulfide bond in the nanocarrier to initiate drug release. The intracellular concentration of GSH is approximately 0.5 to 10 mM.^31^ Hence, such concentration can be attained in an *in vitro* study without difficulty as a GSH-rich cancer cell environment triggers drug release.^32,33^

Two-D nanomaterials have uniquely been considered for biomedical application due to their outstanding electronic, physical, and chemical properties.^34^ As the large surface area of 2D nanomaterials provides adequate area for loading responsive cargo biomolecules, they are favorable materials for stimuli-responsive biomedical applications.^35^ In particular, MoS_2_, WS_2_, MoSe_2_, and WSe_2_ have been studied as a replacement for graphene due to some limitations of graphene such as loss of its native properties when exfoliated using *n*-butyllithium (*n*-BuLi) method, zero band gap, structural defects and so on.^36–40^ As molybdenum (Mo) is an essential element for individual enzymes in cells and S is a universal biological element,^41,42^ MoS_2_ was recently examined to elucidate its potential biomedical applications. Functionalization of the MoS_2_ surface is simple and easy because of the readily available sulfur vacancy on MoS_2_.^43^ The biological applications of MoS_2_-based nanocarriers have also been studied in a variety of therapeutic and diagnostic applications such as bioimaging, drug delivery, gene delivery, phototherapy, combined therapy, theranostic, and biosensing. However, limitations and drawbacks were found in the reported study. An analysis of some future aspects for the long-term development of MoS_2_-based nanocomposites as a potential nanomedicine has highlighted its limits.^44^ Most of the recent studies on MoS_2_ focused on its therapeutic application to mainly target tumors *via* passive release of drug molecules. A stimuli-responsive nanocarrier system with MoS_2_ is therefore essential to achieve an on-demand DDS.^45^

**Fig. 1:**
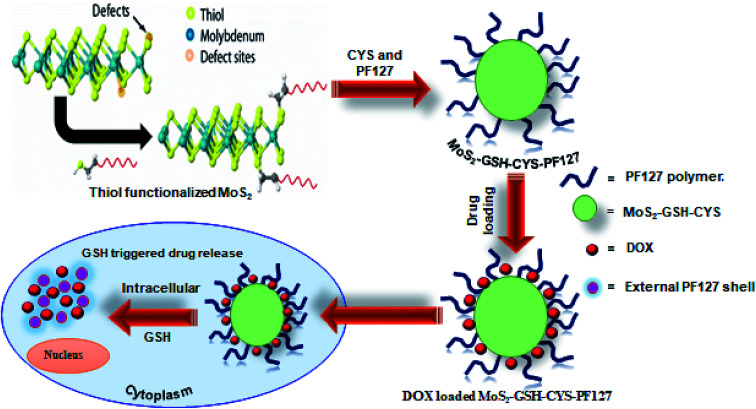
Schematic illustration of the preparation of DOX-loaded MoS_2_–GSH–CYS–PF127 nanocomposites for reduction-sensitive intracellular nucleus drug release.

In this study, a GSH-responsive MoS_2_–GSH–CYS–PF127 nanocomposite was prepared for effective drug delivery in a GSH-rich environment. To prepare these nanocomposites, MoS_2_ was first exfoliated using GSH as a surfactant *via* sonication and this was followed by the addition of a disulfide-containing CYS. Finally, PF127 was introduced to derive the GSH-responsive MoS_2_–GSH–CYS–PF127 nanocomposite. The tailored MoS_2_ nanocomposite system exhibited sensitivity in the GSH environment as confirmed by TEM and DLS. To further evaluate the GSH-sensitive property of the MoS_2_ nanocomposite, the anticancer drug, doxorubicin (DOX), was loaded onto the carrier and its release in a GSH environment was assessed. This GSH reduction-responsive drug release assessment was performed with phosphate buffer pH = 7.4 and GSH = 5 mM. We found that 52% of the drug was released after 72 h. Subsequently, fluorescence microscopic images revealed that a 6 h incubation of the MoS_2_ nanocomposite in HeLa cells effectively killed the cells relative to the 2 h and 4 h periods. To add, the images revealed that the nanocomposite was located in the cell membrane alone. Therefore, this MoS_2_ nanocarrier opens a window for its use as a stimuli-responsive nanocarrier for drug delivery (Fig. 1).

## 2. Experimental section

### 2.1. Materials

MoS_2_ (10–30 μm) was purchased from Rose Mill Company, reduced l-glutathione (GSH), cystamine dihydrochloride (CYS), 3-(4,5-dimethylthiazol-2-yl)-2,5-diphenyltetrazolium bromide (MTT), pluronic F127 (PF127) and biological water were acquired from Sigma-Aldrich. Phosphotungstic acid *N*-hydrate (PTA) were purchased from J.T. Baker, (India), doxorubicin hydrochloride (DOX) were purchased from Cayman Chemical Company (USA), anhydrous dimethyl sulfoxide was obtained from Macron Fine Chemicals. Dulbecco's Modified Eagle Medium (DMEM), penicillin, sodium pyruvate, trypsin and sterilized fetal bovine serum (FBS), were purchased from Gibco (Carlsbad, CA). Human cervical carcinoma (HeLa) cells were obtained from the Bio Resource Collection and Research Center (Hsinchu, Taiwan). Regenerated cellulose tubular membrane with a nominal value of 1000 and 6000–8000 Da was purchased from Orange Scientific. Unless otherwise noted, all other reagents and solvents were obtained from Sigma-Aldrich, Alfa-Aesar, and TCI chemicals. Other reagents, and buffer solution components were of analytical grade. Distilled and deionized water (DI) were used in all experiments. All solutions and reagents were used without further purification.

### 2.2. Characterization of MoS_2_–GSH–CYS–PF127 and DOX-loaded MoS_2_–GSH–CYS–PF127

Transmission electron microscopy (HR-TEM) images were obtained with a Tecnai-F20 FEI-TEM system. Ten μL of the sample solution was placed on a 300 mesh carbon-stabilized, formvar-carbon-coated grid. Following a 2 min adsorption, the sample was washed with distilled water and air-dried. Each grid was then stained with 0.2% (w/v) PTA for 2 min. Excess staining solution was removed, and the sample was allowed to air-dry. After complete drying, grids were observed using HR-TEM and photographed with a CCD camera.

UV-visible images were captured with a Jasco V-730 spectrofluorometer. Particle size and zeta potential of the prepared DOX-loaded nanocomposites were determined by dynamic light scattering (DLS) and their zeta potential was also measured using a zeta potential analyzer. Data are presented as the average of three measurements. The amount of DOX in DOX-loaded MoS_2_–GSH–CYS–PF127 composites was determined by UV-visible spectroscopy using Jasco V-730 spectrofluorometer.

### 2.3. Preparation of MoS_2_–GSH nanoparticles

Eight hundred mg of MoS_2_ and 800 mg of l-GSH (1–1 ratio) were added to 40 mL of dimethyl sulfoxide (DMSO) and probe sonicated for 8–10 h with power density of 650 W. After sonication, the resulting greenish-black solution was allowed to settle for 24 h. After 24 h, centrifugation was conducted to remove the unexfoliated MoS_2_ nanoparticles. The supernatant was collected and dialysis was performed using a 1000 Da-membrane for 4 h to remove excess ligand. Moreover, the anhydrous DMSO assisted MoS_2_ shows good exfoliation with long-term stability. In addition, many researchers revealed that anhydrous DMSO assisted exfoliation can provide effective method to obtained single and few-layer MoS_2_ sheets.^46,47^ After sonication, the resulting greenish-black solution was allowed to settle for 24 h. After 24 h, centrifugation was conducted to remove the unexfoliated MoS_2_ nanoparticles. The supernatant was collected and dialysis was performed using a 1000 Da-membrane for 4 h to remove excess ligand.

### 2.4. Preparation of MoS_2_–GSH–CYS–PF127 nanocomposite

Twenty mg of CYS and PF127 were added to 5 mL of MoS_2_–GSH nanoparticle solution. The nanocomposite was formed during dialysis and this was allowed to proceed to remove excess CYS. Prior to the preparation of the MoS_2_–GSH–CYS–PF127 nanocomposite, MoS_2_–GSH–PF127 and MoS_2_–GSH–CYS were synthesized *via* simple procedures. Twenty milligrams of PF127 was then added to 10 mL of MoS_2_–GSH nanoparticle solution during dialysis to prepare MoS_2_–GSH–PF127. Meanwhile, 20 mg of CYS was added to 10 mL of MoS_2_–GSH nanoparticle solution during dialysis to prepare MoS_2_–GSH–CYS.

### 2.5. Drug loading

The anticancer drug, DOX, was loaded into multifunctional nanocarriers. Briefly, 50 mg of MoS_2_–GSH–CYS–PF127 was added to 50 mL of PBS and 5 mL of DOX solution (5 mg dissolved in 5 mL of DMSO) *via* an ultrasonic bath. The mixture was sealed and stirred for 24 h in the dark at room temperature. Thereafter, the DOX-loaded nanocomposite was dialyzed against PBS to remove unbound DOX molecules.

### 2.6. Drug release experiment in a reduction-sensitive environment

The *in vitro* reduction-sensitive release behavior of the drug was investigated by placing the DOX-loaded MoS_2_–GSH–CYS–PF127 nanocomposite (1.5 mL) in a dialysis bag. The dialysis bag was soaked in 15 mL of PBS with or without 5 mM GSH and placed on shaker set at 37 °C and 180 rpm. At predetermined time intervals, 3 mL of the sample solution was removed and replaced with fresh PBS solution. For reduction-sensitive drug release, an equivalent amount of fresh PBS containing 5 mM GSH was continuously supplemented for 3 days. The amount of cumulative drug release (DOX) was measured *via* absorption at a wavelength of 480 nm.

### 2.7. GSH-responsiveness of DOX-loaded MoS_2_–GSH–CYS–PF127 nanocomposites

The GSH-responsive behavior of DOX-loaded MoS_2_–GSH–CYS–PF127 nanocomposites was investigated in the presence of 5 mM GSH and PBS buffer by DLS. Briefly, GSH (5 mM) was mixed with DOX-loaded MoS_2_–GSH–CYS–PF127 nanocomposites in different environments. The solution was then incubated on a shaking table at 37 °C and changes in size of the nanocomposites were investigated at different time intervals by DLS.

### 2.8. MTT assay

HeLa cell line was cultured at density of 2 × 10^4^ in a 96-well plate and maintained in DMEM (Wisent Inc., USA) supplemented with 10% fetal bovine serum (Wisent Inc.) and 1% sodium pyruvate (Wisent Inc.) at 37 °C in a humidified atmosphere with 5% CO_2_. After a 24 h incubation, the cells were exposed to 200 μg mL^−1^ to 6.25 μg mL^−1^ of MoS_2_–GSH–CYS–PF127, and 40 μg mL^−1^ to 1.25 μg mL^−1^ of free DOX and DOX-loaded MoS_2_–GSH–CYS–PF127 nanocomposites. After a 24 h incubation in a humidified atmosphere, the old medium was removed and fresh medium with MTT (5 mg mL^−1^) was added followed by an additional incubation for 2–4 h for cytotoxic evaluation using an ELISA reader.

### 2.9. Cellular uptake and intracellular localization of DOX

HeLa cells were seeded in a confocal dish (35 mm × 10 mm; Corning Inc., New York, USA) at a density of 1 × 10^5^ cells per well and incubated in DMEM for 24 h. Culture medium was removed and fresh DMEM containing free DOX and DOX-loaded MoS_2_–GSH–CYS–PF127 nanocomposites (5 μg mL^−1^) were added and incubated for 2 h, 4 h, and 6 h in a humidified atmosphere. The cells were washed three times with PBS to remove excess nanoparticles. DAPI in PBS (100 nanomolar) was added to cells prior to incubation at room temperature for 15 min followed by rinsing with PBS and observation by fluorescence microscopy.

## 3. Results and discussion

### 3.1. Particle size and zeta potential

In the present study, we aimed to synthesize a GSH-responsive MoS_2_-based DDS. MoS_2_ was first exfoliated in DMSO *via* sonication and this was followed by the introduction of GSH as a surfactant. A disulfide-containing CYS was then introduced followed by PF127 *via* self-assembly using the dialysis method. The synthesized GSH-responsive nanocarrier system was then examined using a variety of techniques such as UV-visible spectroscopy, TEM, DLS, and zeta potential, and drug release was confirmed by UV-visible spectroscopy and fluorescence microscopy. For MoS_2_–GSH nanoparticles and MoS_2_–GSH–CYS, particle size was ∼61.6 nm and ∼110.2 nm, respectively. However, after the formation of the self-assembled PF127 on the surface of MoS_2_–GSH, the size of the nanocarrier increased to 71.7 nm. Notably, MoS_2_–GSH had a smaller size before CYS loading; however, after loading, particle size was ∼110.2 nm. The greater particle size might be due to the negative charge of MoS_2_–GSH which may have allowed a strong electrostatic interaction with the positive group of CYS.^48,49^ In contrast, PF127 polymer coating reduced the size of the nanocarrier from size of MoS_2_–GSH–CYS particle from 110.2 nm to 82.3 nm.^50,51^ Zeta potential values for MoS_2_–GSH and MoS_2_–GSH–PF127 were −12.2 mV, and −15.7 mV, respectively. As shown in Table 1, the zeta potential of MoS_2_–GSH–CYS and MoS_2_–GSH–CYS–PF127 increased toward positive values of 0.5 mV and 1.7 mV.

**Table 1:** Hydrodynamic size and zeta potential values of MoS_2_–GSH, MoS_2_–GSH–PF127, MoS_2_–GSH–CYS, MoS_2_–GSH–CYS, and MoS_2_–GSH–CYS–PF127

System	Size (nm)	Zeta (mV)
MoS_2_–GSH	61.16	−12.16
MoS_2_–GSH–PF127	71.7	−15.7
MoS_2_–GSH–CYS	110.2	0.5
MoS_2_–GSH–CYS–PF127	82.3	1.7

### 3.2. UV-visible spectroscopy

**Fig. 2:**
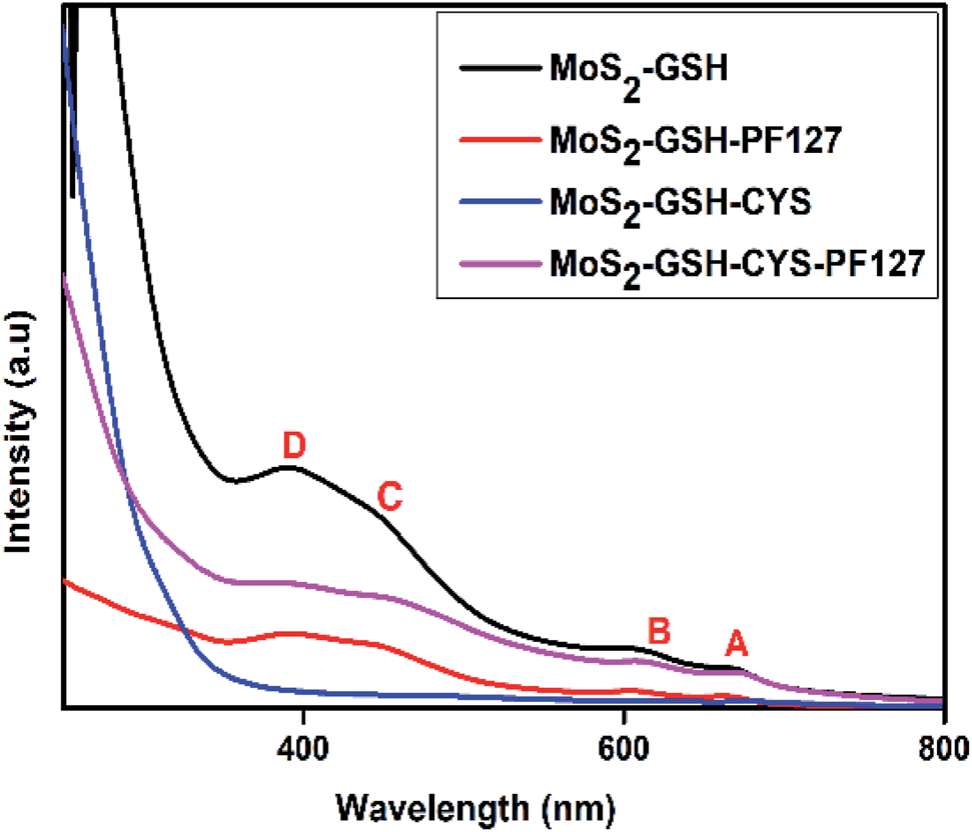
UV-visible spectra of MoS_2_–GSH, MoS_2_–GSH–PF127, MoS_2_–GSH–CYS, MoS_2_–GSH–CYS, and MoS_2_–GSH–CYS–PF127.

The synthesized MoS_2_ nanocarrier was confirmed by UV-visible spectroscopy and the spectra for MoS_2_–GSH, MoS_2_–GSH–PF127, MoS_2_–GSH–CYS, and MoS_2_–GSH–CYS–PF127 are shown in Fig. 2. For MoS_2_–GSH, four characteristic peaks were observed at 669 nm (A), 606 nm (B), 445 nm (C), and 391 nm (D), which agree with the reported values.^52^ The absorption peaks, namely A and B, were due to direct excitonic transitions at the *K*-point with energy difference caused by valence band spin–orbital coupling. Peaks C and D were assigned to the direct excitonic transition of the *M*-points between higher densities of state and region of the band structure. This study confirms the presence of synthesized exfoliated MoS_2_ samples exhibits 2H MoS_2_.

After PF127 was coated onto the MoS_2_–GSH system, the wavelength decreased to 664 nm, 605 nm, 444 nm, and 390 nm. As the observed blue shift is usually caused by H-aggregates, this result suggests that the amphiphilic polymer results in dense parallel-aggregates of MoS_2_ in the core of PF127. The wavelengths depicting the characteristic maximum absorption peaks shifted from 669 nm (A), 606 nm (B), 445 nm (C), and 391 nm (D) to 675 nm, 617 nm, 457 nm, and 397 nm for MoS_2_–GSH–CYS, and 672 nm, 611 nm, 446 nm, and 395 nm for MoS_2_–GSH–CYS–PF127. As the observed red shift is usually caused by J aggregates, this result suggests that CYS induce the nanoparticle aggregation of MoS_2_–GSH and MoS_2_–GSH–PF127, which also proved in the DLS results (Table 2).^49^

**Table 2:** 2H MoS_2_ UV-visible maximum absorbance excitonic peaks of MoS_2_–GSH, MoS_2_–GSH–PF127, MoS_2_–GSH–CYS, MoS_2_–GSH–CYS, and MoS_2_–GSH–CYS–PF127

UV-excitonic peaks	A (nm)	B (nm)	C (nm)	D (nm)
MoS_2_–GSH	669	606	445	391
MoS_2_–GSH–PF127	664	605	444	390
MoS_2_–GSH–CYS	675	617	457	397
MoS_2_–GSH–CYS–PF127	672	611	446	395

### 3.3. Transmission electron spectroscopy (TEM)

**Fig. 3:**
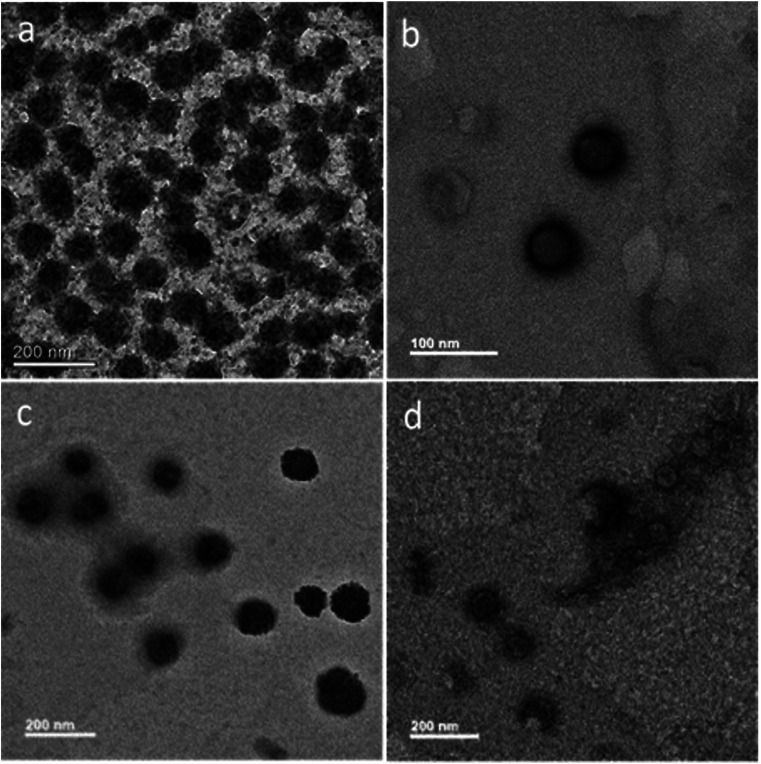
HR-TEM images of negative staining with phosphotungstic acid (PTA): (a) MoS_2_–GSH, (b) MoS_2_–GSH–PF127, (c) MoS_2_–GSH–CYS, and (d) MoS_2_–GSH–CYS–PF127.

PTA was used as the negative staining agent, thereby allowing a contrast between background and the MoS_2_ core. Each sample was stained with 0.2% (w/v) PTA for 2 min and the morphology was investigated by TEM. As shown in Fig. 3a, MoS_2_–GSH nanoparticle had a spherical shape and a size of ∼80–90 nm; this result was similar to that achieved by DLS. As shown in Fig. 3b, MoS_2_–GSH–PF127 displayed a homogenous and core shell that had a spherical structure and size of ∼50–60 nm. However, when CYS was coated on the surface of MoS_2_–GSH, the morphology changed to a core shell structure and its size increased to ∼100–150 nm. These results also support the DLS results (Fig. 3c).^48^ MoS_2_–GSH–CYS–PF127 (Fig. 3d) had a core shell structure with additional small and larger-sized aggregate particles that resembled the nanocomposites.

### 3.4. Determination of drug loading of MoS_2_–GSH–CYS–PF127 nanocomposites

To determine drug loading and encapsulation efficiency, 5 mg of the freeze-dried nanocomposites were dispersed in 5 mL of PBS. The loading capacity of the MoS_2_ nanocomposites were measured by UV-vis at an absorbance wavelength of 480 nm. Percent drug loading and encapsulation efficiency were calculated using the following equations:

DOX concentration was calculated as:
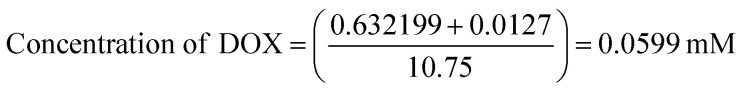

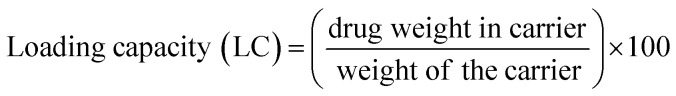

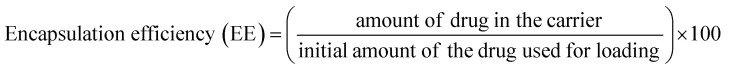


The calculated drug loading capacity and encapsulation efficiency were 51.36% and 56%, respectively.

### 3.5. UV-visible spectroscopy, particle size, zeta potential, GSH reduction sensitivity, and cumulative drug release studies of DOX-loaded MoS_2_–GSH–CYS–PF127

**Fig. 4:**
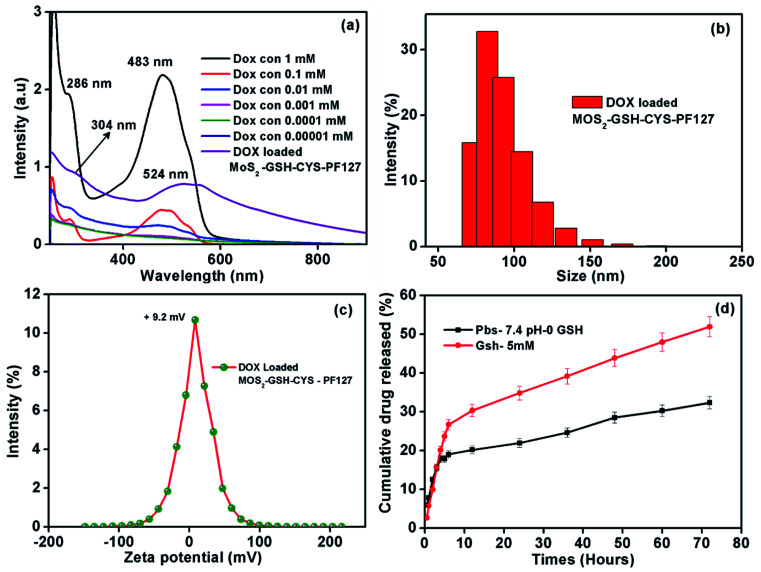
(a) UV-visible spectra of the different concentrations of DOX and DOX-loaded MoS_2_–GSH–CYS–PF127 composite used to construct the calibration curve. (b) Hydrodynamic size of MoS_2_–GSH–CYS–PF127. (c) Zeta potential values of DOX-loaded MoS_2_–GSH–CYS–PF127. (d) Cumulative drug release profile (%) of DOX from MoS_2_–GSH–CYS–PF127 at 37 °C with pH 7.4 PBS and 5 mM GSH.

As shown in Fig. 4a, the characteristic maximum absorbance for DOX was ∼286 nm and 483 nm, which are values similar to previous reports.^53,54^ Furthermore, the DOX peak in the drug-loaded MoS_2_–GSH–CYS–PF127 was ∼302 and 524; these were observed to shift from the native DOX peaks of ∼286 and 483. This shifting might be due to the electron donor–acceptor interaction between DOX and MoS_2_ nanocomposites.^55,56^ The size and zeta potential of the drug-loaded MoS_2_ nanocomposites were significantly increased (Table 2). Before loading, the MoS_2_ nanocomposites had a size of ∼82.3 nm and zeta potential value of ∼1.7 mV. After DOX loading, the size of the MoS_2_ nanocomposites was ∼102 and zeta potential value was 9.4 mV. This result indicates that the nanoparticles were nearly electrically neutral which is beneficial for prolonged circulation in blood without the possibility of elimination.^57^ The stability of DOX-loaded MoS_2_–GSH–CYS–PF127 has been evaluated for one month, and the size of drug carrier showed around 99.7 nm (Table 3), which also proved that the PF127 has successfully covered on the surface of drug carrier. The GSH sensitivity of the drug-loaded MoS_2_ nanocomposites was explored in the presence and absence of 5 mM GSH in PBS buffer by measuring the changes in particle size at different time intervals. As shown in Table 3, the particle size of the DOX-loaded MoS_2_–GSH–CYS–PF27 increased from 102 to 261 nm in 24 h, 479 nm in 48 h, and 778 nm in 72 h in the presence of GSH. This is due to the cleavage of the disulfide linkage in the core of the MoS_2_ nanocomposites, thereby leading to larger particle sizes. In contrast, dramatic size changes were not found in drug-loaded MoS_2_ nanocomposites in the absence of GSH.

**Table 3:** Particle size and zeta potential values of MoS_2_–GSH–CYS–PF127 and DOX-loaded MoS_2_–GSH–CYS–PF127 before the drug release

System	Particles size (nm ± 2)	Zeta potential (mV ± 2)
MoS_2_–GSH–CYS–PF127	82.3	1.7
DOX-loaded MoS_2_–GSH–CYS–PF127	102	9.4
DOX-loaded MoS_2_–GSH–CYS–PF127 (I month)	99.7	9.3

By using the dialysis method, we demonstrated the release of DOX from MoS_2_–GSH–CYS–PF127 nanocomposites at 37 °C in PBS buffer (pH 7.4) in the presence and absence of 5 mM GSH. The DOX solution was removed from the drug release setup and UV was measured at predetermined intervals for 72 h. As shown in Fig. 4d, the 5 mM GSH-containing MoS_2_ nanocomposites released more DOX (52%) relative to the nanocomposites without GSH (32%). Due to the disulfide reducing CYS present in the core of the MoS_2_ nanocomposites, introducing GSH breaks the disulfide bond, ultimately leading to drug release.^58–60^ The introduction of 5 mM GSH to drug loaded MoS_2_ system effectively reduce the disulphide bond of cystamine results the dissociation of ligands (GSH and CYS) and recombination of ligands (GSH and CYS) on the surface of MoS_2_. In addition, the disruption of cysteamine positive cystamine on the surface easily to make the aggregation of negative charge of MoS_2_ with surface positive charge cystamine and then finally leading to increase the size in DLS measurement (Table 4). However, the particle size of DOX-loaded MoS_2_–GSH–CYS–PF127 is increased while absence of GSH environment which may occurred due to instability of PF127 during drug release environment. The fresh phosphate buffer has been added and make dilution of DOX-loaded MoS_2_–GSH–CYS–PF127 solution and resulted the particle size slightly increased at 48 and 72 hours.^61^

**Table 4:** Particle sizes of DOX-loaded MoS_2_–GSH–CYS–PF127 in the presence and absence of a GSH environment after the drug release in different time point

Time (h)	Particle size (nm ± 2) presence of 5 mM GSH	Particle size (nm ± 2) PBS buffer, absence of GSH
24	261	104
48	479	139
72	778	284

### 3.6. *In vitro* cytotoxicity of MoS_2_–GSH–CYS–PF127, free DOX, and DOX-loaded MoS_2_–GSH–CYS–PF127 nanocomposites

**Fig. 5:**
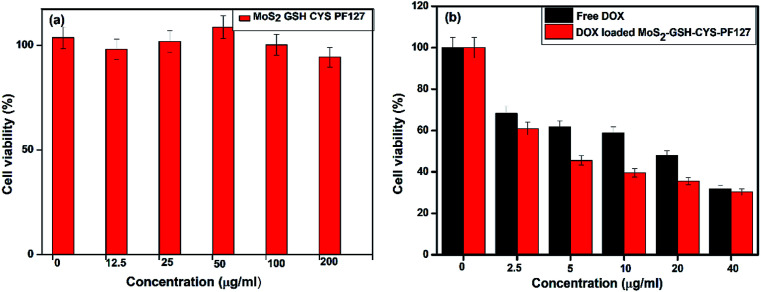
(a) MTT assay for MoS_2_–GSH–CYS–PF127, (b) DOX-loaded MoS_2_ nanocomposite, and free DOX at several concentrations.

The cytotoxicity of nanomaterials used in DDSs is the most important factor that can reduce their adverse side effects. Hence, the cytotoxicity of the synthesized MoS_2_–GSH–CYS–PF127 was evaluated by the MTT assay. Prior to drug loading, the MoS_2_ nanocomposite did not exhibit any significant toxicity and almost 95% of the cells were viable even when the concentration of the nanocarrier reached 200 μg mL^−1^; this result proved the biocompatibility of the nanocomposite. As demonstrating the sensitivity of drug release under reduced GSH environment is important, different concentrations of DOX-loaded MoS_2_–GSH–CYS–PF127 were incubated with HeLa cells for 24 h to evaluate drug release. The results were then assessed using the MTT assay. Concentration-dependent toxicity was observed in DOX-loaded MoS_2_–GSH–CYS–PF127. Moreover, a maximum cell death of almost 65% was observed at 40 μg mL^−1^. Interestingly, the cell death ratio of DOX-loaded MoS_2_–GSH–CYS–PF127 was almost equal to that of free DOX. These results proved the potential of GSH to induce drug release from the MoS_2_ nanocomposite system and the capability of this nanocomposite as a drug carrier. To differentiate between the toxicity of the nanocomposite and drug, the cytotoxicity of the leading MoS_2_–GSH–CYS–PF127 nanocomposites was initially resolved prior to the addition of free DOX and DOX-loaded nanoparticles. The cytotoxicity of free DOX and DOX-loaded MoS_2_–GSH–CYS–PF127 was assessed using HeLa cells at several concentrations after a 24 h treatment. This assured that MoS_2_–GSH–CYS–PF127 could be used as a drug carrier. Herein, 95% of HeLa cells were viable after a 24 h incubation, even at the high concentration of 200 μg mL^−1^. From 100 μg mL^−1^ to 12.5 μg mL^−1^, cell viability reached more than 100% as shown in Fig. 5a; (the free nanoparticle concentrations were 200, 100, 50, 25, 12.5, and 0 μg mL^−1^). We also found that DOX and DOX-loaded MoS_2_–GSH–CYS–PF127 displayed low toxicity at the concentrations tested (free DOX and DOX-loaded nanocomposites: 40, 20, 10, 5, 2.5, and 0 μg mL^−1^). As shown in Fig. 5b, HeLa cells were treated with free DOX and DOX-loaded MoS_2_–GSH–CYS–PF127 nanocomposites for 24 h. The HeLa cells incubated with DOX loaded MoS_2_–GSH–CYS–PF127 showed 38% toxicity at drug concentration of 40 μg, which indicated that the successfully released of drug from carrier and caused the cell death. Moreover, DOX-loaded MoS_2_–GSH–CYS–PF127 nanocomposites exhibited lower cytotoxicity than free DOX. As mentioned above, free DOX is slightly more cytotoxic than DOX-loaded MoS_2_–GSH–CYS–PF127 toward HeLa cells. This is because of the low molecular weight of DOX enables its easy diffusion into the cell without the drug release process.^62^

### 3.7. Cellular uptake and intracellular release of DOX-loaded MoS_2_–GSH–CYS–PF127 nanocomposites

**Fig. 6:**
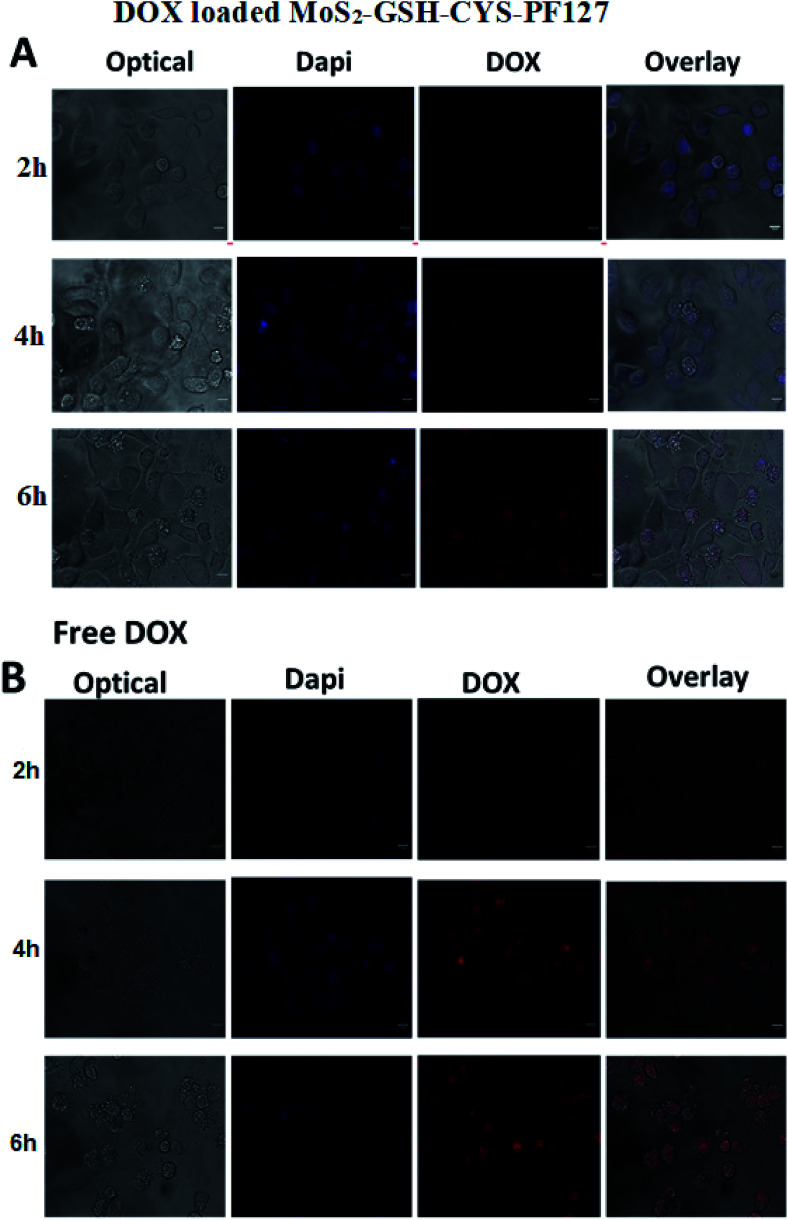
(A) Internalization and retention of DOX-loaded MoS_2_–GSH–CYS–PF127 in HeLa cells 2 h, 4 h, and 6 h following the removal of the DOX formulations as measured by confocal microscopy. (B) Internalization and retention of free DOX in HeLa cells 2 h, 4 h, and 6 h after the removal of DOX-loaded MoS_2_–GSH–CYS–PF127 nanocomposites as measured by confocal microscopy. Scale bar represents 10 μm.

The results of the *in vitro* drug release studies inspired us to investigate the cellular uptake and intracellular distribution of free DOX and DOX-loaded MoS_2_–GSH–CYS–PF127 nanocomposites. Hence, we employed HeLa cells for the evaluation and a fluorescence microscope for visualization. The concentration of free DOX and DOX in the MoS_2_ carrier was fixed to 5 μg mL^−1^. Fig. 6A shows the fluorescent microscopic images of HeLa cells incubated with DOX-loaded MoS_2_–GSH–CYS–PF127 nanocomposites. These cells displayed a faint red DOX fluorescence within the cell membrane or cytoplasm of cells at the beginning of incubation (*i.e.*, 2–4 h). However, after 6 h of incubating the MoS_2_ carrier with HeLa cells, a strong red fluorescence was observed in the nucleus of HeLa cells depicting DOX release from the carrier, followed by internalization into the nucleus. Drug-loaded MoS_2_–GSH–CYS–PF127 nanocomposites efficiently released the drug under the intracellular GSH-rich condition and the released drug subsequently diffused into the nuclei of HeLa cells. The internalization of free DOX by HeLa cells also resulted in a strong red color and this was even evident at 2 h (Fig. 6B). However, a higher number of dead cells was observed with free DOX than DOX-loaded MoS_2_ nanocomposites.^63,64^

## 4. Conclusion

In this study, we prepared a DOX-loaded PF127 self-assembled MoS_2_ nanocomposite and demonstrated its ability to release the transported drug under reduced GSH conditions. To construct the GSH-sensitive MoS_2_ nanocomposite, we introduced a disulfide-containing CYS to the exfoliated MoS_2_ prior to introducing PF127. By TEM imaging, the synthesized MoS_2_ nanocomposite was found to have a spherical shape and size of ∼82.3 nm while the DOX-loaded MoS_2_ nanocomposite had a size of 102 nm. In 5 mM GSH, the MoS_2_ nanocomposite released 52% of the transported drug in 72 h. In addition, by performing an MTT assay, the biocompatibility of the nanocomposite was confirmed. The DOX-loaded nanocomposite was demonstrated to be highly toxic due to the release of DOX in the GSH-rich cancer cell environment. The DOX release behavior of the carrier was further supported by images obtained *via* fluorescence microscopy. Compared to 2 h, after 6 h of incubation, the DOX-loaded nanocomposite effectively released DOX which was internalized into the nucleus. After a 4 h incubation, DOX was only found in the cell membrane and cytoplasm.

## Conflicts of interest

There are no conflicts to declare.

## Supplementary Material
